# Differential susceptibility of human motor neurons to infection with Usutu and West Nile virus

**DOI:** 10.1186/s12974-024-03228-y

**Published:** 2024-09-27

**Authors:** Eleanor M. Marshall, Lisa Bauer, Tessa Nelemans, Syriam Sooksawasdi Na Ayudhya, Feline Benavides, Kristina Lanko, Femke M. S. de Vrij, Steven A. Kushner, Marion Koopmans, Debby van Riel, Barry Rockx

**Affiliations:** 1 Department of Viroscience, Erasmus Medical Center, Dr. Molewaterplein 40, Rotterdam, 3015 GD The Netherlands; 2 Department of Medical Microbiology, Leiden University Medical Centre, Leiden, the Netherlands; 3 Department of Clinical Genetics, Erasmus Medical Center, Rotterdam, The Netherlands; 4 Department of Psychiatry, Erasmus Medical Center, Rotterdam, The Netherlands; 5 Department of Psychiatry, Columbia University Medical Center, New York, NY USA; 6 Faculty of Veterinary Science, Prince of Songkla University, Songkhla, Thailand

**Keywords:** Flavivirus, West Nile virus, Usutu virus, Neuroinvasion, West Nile acute flaccid paralysis, Motor neurons, Antiviral response, Interferon

## Abstract

West Nile virus (WNV) and Usutu virus (USUV) are closely related flaviviruses with differing capacities to cause neurological disease in humans. WNV is thought to use a transneural route of neuroinvasion along motor neurons and causes severe motor deficits. The potential for use of transneural routes of neuroinvasion by USUV has not been investigated experimentally, and evidence from the few clinical case reports of USUV-associated neuroinvasive disease is lacking. We hypothesised that, compared with WNV, USUV is less able to infect motor neurons, and therefore determined the susceptibility of human induced pluripotent stem cell (iPSC)-derived spinal cord motor neurons to infection. Both viruses could grow to high titres in iPSC-derived neural cultures. However, USUV could not productively infect motor neurons due to restriction by the antiviral response, which was not induced upon WNV infection. Inhibition of the antiviral response allowed for widespread infection and transportation of USUV along motor neurons within a compartmented culture system. These results show a stark difference in the ability of these two viruses to evade initiation of intrinsic antiviral immunity. Our data suggests that USUV cannot infect motor neurons in healthy individuals but in case of immunodeficiency may pose a risk for motor-related neurological disease and transneural invasion.

**Supplementary Information:**

The online version contains supplementary material available at 10.1186/s12974-024-03228-y.

## Brief summary

West Nile virus, but not Usutu virus, can productively infect human motor neurons as a possible route of neuroinvasion.

**Supplementary Information:**

The online version contains supplementary material available at 10.1186/s12974-024-03228-y.

## Background

West Nile virus (WNV) is a mosquito-borne flavivirus that can cause severe neurological disease in humans and animals. Since its introduction into the United States of America in 1999, WNV has become one of the leading causes of viral encephalitis in North America and increasingly in Europe, with thousands of cases of disease and hundreds of WNV-related deaths reported each transmission season [1–4]. Usutu virus (USUV) is closely related to WNV, and both viruses co-circulate in Europe, but the risk to human health posed by USUV is not well understood. Only sporadic cases of USUV-associated neurological disease have been reported [5], despite evidence indicating a higher seroprevalence of USUV compared to WNV in regions where both viruses are enzootic and co-circulating [6]. The comparatively low incidence and severity of disease caused by infection with USUV suggests a reduced capacity of this virus to induce neuroinvasive disease when compared with WNV.

The three overarching presentations of West Nile neuroinvasive disease in humans are West Nile Meningitis, West Nile Encephalitis and West Nile Acute Flaccid Paralysis (AFP). Severe motor dysfunction is a hallmark of West Nile AFP, with viral presence and damage to motor neurons in the anterior horn of the spinal cord identified post-mortem [7–13]. WNV is thought to use numerous modes of neuroinvasion to gain access to the central nervous system (CNS), including an ascending route via motor neurons into the spinal cord and subsequently to the basal regions of the brain [14, 15]. The susceptibility of motor neurons to infection with USUV, as a prerequisite for transneural invasion of the CNS, has not been investigated in vitro, and clinical case reports appear to indicate a reduced severity, progression and persistence of motor-related dysfunction [5, 6, 16–21] compared with WNV. Therefore, we hypothesised that USUV is less able to infect motor neurons and, as a result, is less likely to induce severe, sustained motor impairment and neurological disease.

In the past, the study of viral neuropathogenesis has been hampered by a lack of relevant model systems. However, the increased application of human induced pluripotent stem cells (iPSCs) to disease modelling in recent years has begun to bridge the gap between simple in vitro cell lines and the complex microenvironment of the human peripheral and central nervous system [22]. Here, to address our hypothesis, we employed two iPSC-derived neuron culture systems to model the human brain and spinal cord motor neurons. We compared the susceptibility of these cortical and spinal cord motor neuron models to infection with USUV and WNV and investigated the induction of the host intrinsic response to identify the key factors underlying the pattern of susceptibility observed.

## Materials and methods

### Human iPSC acquisition and culture

Human induced pluripotent stem cells (iPSCs) [WTC-11 provided by Bruce R. Conklin, the Gladstone Institutes and UCSF Institute, San Francisco, CA, USA] were used to generate neuronal determinant neurogenin-2 (Ngn2) neurons, astrocytes and spinal cord motor neurons (sMNs) (Fig. S1). iPSCs were maintained in iPSC medium (Table 1.) at 37 °C and 5% CO2. Accutase (Life Technologies) was used for dissociation during passaging and cells were seeded on Matrigel (Corning, 10 μl/ml) coated 6-well plates. For coating, Matrigel was diluted in Knock-Out Dulbecco’s Modified Eagle Medium (KO DMEM; ThermoFisher Scientific) and plates were incubated for a minimum of 1 h at 37 °C.

**Table 1:** Components of basal media used for differentiation and iPSC-derived cultures

Name	Reagents and final concentrations	Manufacturer
iPSC medium	Stemflex medium	Thermofisher Scientific
	100 IU/ml penicillin	Lonza
	100 μg/ml streptomycin	Lonza
	1X Revitacell	Thermofisher Scientific
Differentiation medium	Advanced DMEM/F12 medium	Thermofisher Scientific
	100 IU/ml penicillin	Lonza
	100 μg/ml streptomycin	Lonza
	0.1mM non-essential amino acids	Lonza
	1x N2 supplement	Thermofisher Scientific
	10ng/ml Human Recombinant neurotrophic factor (NT3)	Stemcell Technologies
	10ng/ml brain-derived neurotrophic factor (BDNF)	Peprotech
	200ng/ml laminin	Corning
	4 μg/ml Doxycycline (DOX)	Sigma
Ngn2 neuron-astrocyte co-culture medium	Neurobasal medium	Thermofisher Scientific
	100 IU/ml penicillin	Lonza
	100 μg/ml streptomycin	Lonza
	2mM glutamine	Lonza
	2% B27 supplement without RA	Thermofisher Scientific
	10ng/ml Human Recombinant neurotrophic factor (NT3)	Stemcell Technologies
	10ng/ml brain-derived neurotrophic factor (BDNF)	Peprotech
	4 μg/ml Doxycycline (DOX)	Sigma
sMN basal medium	DMEM: Ham’s F12	Lonza
	Neurobasal medium	Thermofisher Scientific
	100 IU/ml penicillin	Lonza
	100 μg/ml streptomycin	Lonza
	1 x N2 supplement	Thermofisher Scientific
	1 x B27 supplement without RA	Thermofisher Scientific
	1 μl/ml 2-Mercaptoethanol	Thermofisher Scientific
	0.5μM ascorbic acid	Sigma

### Differentiation of human iPSCs to Ngn2 neural co-cultures

Stably transduced WTC-11 Ngn2 iPSCs were directly differentiated into excitatory cortical layer 2/3 neurons by overexpression of neurogenin-2 (Ngn2) [23–25] using an adapted protocol for inducible overexpression in the presence of doxycycline, as previously described [23]. In short, on day 0, coverslips were coated with poly-L-ornithine (Sigma, 100 μg/ml) for 1 h at room temperature in the dark. Coverslips were then washed 3 times with sterile deionised water and air-dried for 30 min. A droplet of Matrigel was placed in the middle of the coverslip and incubated for 1 h at 37 °C then removed. A 50 μl droplet of Ngn2-iPSC cell suspension was then placed on a coated coverslip and incubated for 30 min at 37 °C 5% CO_2_ to promote cell attachment. After attachment, wells were filled with iPSC medium supplemented with doxycycline (4 μg/ml) (Table 1.). The next day (day 1), medium was refreshed with differentiation medium (Table 1.). To promote synaptic maturation, iPSC-derived astrocytes were added to the culture in a 1:1 ratio [26]. Neural progenitor cells derived from the WTC-11 human iPSC line were differentiated into astrocytes in 4 weeks using a combination of LIF and BMP4 as previously described [25]. The neuron-astrocyte co-culture medium was refreshed the day after with Ngn2 medium (Table 1.). During the differentiation and maturation, half of the medium was refreshed every other day. After 21 days the neural co-cultures were considered mature and used in experiments.

### Differentiation of human iPSCs to spinal cord motor neurons

iPSCs were differentiated into sMNs as previously described [27, 28] and expressed the expected motor neuron-specific markers (Fig. S2). Briefly; on day 0, iPSCs were dissociated using Collagenase type IV (Thermofisher Scientific) to form embryoid bodies in suspension, which were cultured for 2 days at 37 °C in sMN basal medium (Table 1.) supplemented with 5μM Y-27632 (Merck Millipore), 40μM SB431542 (Tocris Bioscience), 0.2μM LDN (Stemgent) and 3μM CHIR99021 (Tocris Bioscience). On day 2 and 5, medium was changed to sMN basal medium supplemented with 0.1μM retinoic acid (Sigma) and 500nM SAG (Merck Millipore). On day 7, medium was changed to sMN basal medium supplemented with retinoic acid, SAG, 10ng/ml BDNF (Peprotech) and 10ng/ml GDNF (Peprotech). On day 9, medium was changed to sMN basal medium supplemented with retinoic acid, SAG, BDNF, GDNF and 10μM DAPT (Tocris Bioscience). On day 10, embryoid bodies were dissociated to single cells at 37 °C using 0.05% trypsin (Gibco). Cells were then plated at a density of 6.5 × 10^4^ cells per well in 24 well plates containing glass coverslips, precoated with poly-L-Ornithine (Sigma) and Matrigel, in day 9 medium. On day 11, half of the medium in each well was replaced with fresh day 9 medium. On day 14, half of the medium in each well was replaced with sMN basal medium supplemented with BDNF, GDNF and 20μM DAPT (Tocris Bioscience). On day 16, half of the medium in each well was replaced with sMN basal medium supplemented with BDNF, GDNF, 10ng/ml CNTF (Preprotech) and 20μM DAPT. From day 17 onwards, medium was replaced with sMN basal medium supplemented with BDNF, GDNF and CNTF every 2–3 days.

### Seeding of compartmented culture device

On day 10 of sMN differentiation, 4.5 × 10^4^ cells in 40 μl of day 9 medium were seeded in the poly-L-Ornithine and Matrigel-coated cell body compartment of an Omega4 compartmented culture device (Enuvio) and left to adhere for 1 h at 37 °C, then topped up to 140 μl. 100 μl of medium was placed in the distal axon compartment of the compartmented culture device, and differentiation continued as stated above. Compartmented devices were used between 2 and 4 weeks post-seeding.

### Virus strains and culturing

Viruses were grown and passaged on Vero cells (African green monkey kidney epithelial cells, ATCC CCL-81) at an MOI of 0.01 for 5–6 days in Dulbecco’s modified Eagle’s medium (DMEM; Lonza) with 2% heat-inactivated foetal bovine serum (Sigma-Aldrich), 100U/ml penicillin, 100μg/ml streptomycin (Lonza) and 2mM L-glutamine (Lonza). Supernatant was harvested and spun at 4000xg for 10 min to remove cell debris. Spun supernatant was aliquotted and frozen at -80 °C. The virus strains used in this study were USUV (lineage Africa 3, GenBank accession MH891847.1, EVAg 011 V-02153, isolated in 2016 from *Turdus merula*) and WNV (lineage 2, GenBank accession OP762595.1, EVAg 010 V-04311, isolated in 2020 from *Phylloscopus collybita*). The USUV and WNV strains used represent prevalent strains circulating in Europe [29, 30], thereby modelling the risk situation in Europe. All virus stocks were sequenced and used at passage 3.

### USUV and WNV infection of iPSC-derived neuronal cultures and inhibition of IFN response

iPSC-derived Ngn2 neural co-cultures and sMN cultures were used between days 21–35. When stated, iPSC-derived sMNs were pre-treated with 8μM of Ruxolitinib (reconstituted in DMSO to a stock concentration of 10mM, SelleckChem), a JAK1/2 inhibitor, or 5 μg/ml of a neutralising anti-Human IFN-Alpha/Beta Receptor Chain 2 antibody (Clone MMHAR-2, PBL assay science), diluted in day 17 medium, at 37 °C for 2 h prior to infection. Treatments were maintained throughout the infection and subsequent culture period. For infection, all medium was removed before addition of virus inoculum diluted in day 17 medium, for sMNs, or in Ngn2 medium, for Ngn2 neural co-cultures, to an MOI of 0.01 or 1. For determination of ISG responses in sMNs, an MOI of 10 was used to ensure exposure of all cells to virus, thereby synchronising infection to increase the chances of observing small differences and reduce the variability potentially introduced by non-exposed/non-infected cells. Plates were returned to the incubator at 37 °C for 1 h, then the virus inoculum was removed before addition of fresh medium. Supernatant was removed and refreshed at the specified time points. The harvested supernatants were stored at -80 °C for titration or RNA isolation. For infection of compartmented culture devices, fluidic isolation of the distal axon compartment was maintained at all times throughout infection and subsequent incubation.

### Tissue titration

Tenfold serial dilutions of supernatants were inoculated onto a monolayer of Vero cells in a 96-well plate (2.3 × 10^4^ cells/well). Cytopathic effect (CPE) was used as the readout and determined at 6 days post-infection (dpi), and virus titres were calculated as the 50% tissue culture infective dose (TCID50) using the Spearman-Kärber method [31]. An initial 1:10 dilution of supernatant resulted in a detection limit of 31.6 TCID50/ml.

### RNA isolation and real-time reverse transcription quantitative PCR for quantification of virus

Sample supernatants or cell lysates in MagnaPure lysis buffer were incubated with Agencourt AMPure XP (Berckman Coulter) magnetic beads in a 96-well plate. The plate was then placed on a DynaMag^TM^-96 magnetic block (Invitrogen) and supernatant was removed. The beads were washed 3 times with 70% ethanol whilst still on the magnetic block and then left to air dry. The plate was removed from the magnetic block and the beads were resuspended in de-ionised water to elute the isolated RNA.

A real-time TaqMan™ assay was performed using the Applied Biosystems 7500 real-time PCR system (Thermo Fisher Scientific). Primer/probe mix (Table 2.) was diluted in TaqMan™ fast virus 1-Step Master Mix and topped up with de-ionised water to a final volume of 12 μl before addition of 8 μl sample RNA. The following program was used: 5 min 50 °C, 20 sec 95 °C and 45 cycles of 3s 95 °C and 30 sec 60 °C. Samples were compared to a standard curve of virus stock dilutions to acquire a TCID50 equivalent/ml.

**Table 2:** Sequences of USUV and WNV primers and probes

Virus	Forward primer	Reverse primer	Probe
USUV	CAAAGCTGGACAGACATCCCTTAC	CGTAGATGTTTTCAGCCCACGT	AAGACATATGGTGTGGAAGCCTGATAGGCA
WNV	CCACCGGAAGTTGAGTAGACG	TTTGGTCACCCAGTCCTCCT	TGCTGCTGCCTGCGGCTCAACCC

### RNA isolation and real-time quantitative PCR for quantification of host interferon response

Supernatant was removed and cells were gently washed with PBS, then immediately lysed with TriPure™ (Sigma-Aldrich) at the specified time point. RNA was extracted from the intracellular lysates using 5PRIME Phase Lock Gel tubes (Quantabio) for phase separation with chloroform. Next, RNA was precipitated from the aqueous phase using isopropanol. The RNA was then reverse-transcribed into cDNA using the RevertAid H Minus reverse transcriptase (Thermo Scientific) and a combination of random hexamers and oligo(dT)20 primer. To measure the host interferon response a real-time quantitative PCR was performed with iQ SYBR Green Supermix (Biorad). The samples were run in a CFX384 Touch real-time PCR detection system (Bio-Rad) using the following program: 3 min at 95 °C and 30 s at 60 °C, followed by 40 cycles of 10 s at 95 °C, 10 s at 60 °C and 30 s at 72 °C, and ending with 10 s at 95 °C and melt curve analysis using a temperature gradient from 60 °C to 95 °C with a 0.5 °C increment. Gene expression was quantified by the standard curve method using a standard dilution series of the samples in the qPCR to determine the mRNA levels as arbitrary units. mRNA levels were then normalised to RPL13a, and the fold change over the mock was calculated. The primers used for qPCR of host genes are listed in Table 3.

**Table 3:** Sequences of primers used for RT-qPCR of host genes

Gene	Forward primer	Reverse primer
IFNβ	TGCTCCAGAACATCTTTG	GATGGTTTATCTGATGATAGAC
IFIT2	GGACCAAAGTCTAAATAGGG	GGCACTTGAATTCACATTG
ISG15	TCCTGGTGAGGAATAACAAGGG	GTCAGCCAGAACAGGTCGTC
RSAD2	TTGGACATTCTCGCTATCTCCT	AGTGCTTTGATCTGTTCCGTC
RPL13a	AAGGTGGTGGTCGTACGCTGTG	CGGGAAGGGTTGGTGTTCATCC

### Immunofluorescent staining

Cells were fixed at the specified time points by incubating for 30 min in 10% formalin. In the compartmented culture system, cells were fixed for 2 h to ensure inactivation of virus in the microchannels. Fixed cells were permeabilised with 0.5% triton (Sigma) diluted in PBS for 30 min. Cells were blocked with 5% bovine serum albumin (BSA; Aurion) for 1 h before incubation with primary antibodies diluted in PBS with 2% BSA overnight at 4 °C. Cells were gently washed three times in PBS then incubated with secondary antibodies for 1 h at room temperature in the dark. Cells were gently washed three times then incubated with Hoechst (1:1000, Invitrogen) for 20 min at room temperature in the dark. Images were obtained using a Zeiss LSM 700 laser scanning microscope.

The primary antibodies used in this study were: mouse anti-flavivirus envelope protein (1:250, D1-4G2-4-15 hybridoma; ATCC, USA), rabbit anti-MAP2 (1:200, Millipore), guinea pig anti-MAP2 (1:200, Synaptic systems), rabbit anti-NF200 (1:200, Sigma Aldrich) and rabbit anti-GFAP (1:200, Millipore). The secondary antibodies used in this study were: donkey anti-mouse AF488 (1:250, Invitrogen), donkey anti-rabbit AF488 (1:200, Invitrogen), donkey anti-rabbit AF555 (1:200, Invitrogen), donkey anti-guinea pig AF647 (1:200, Invitrogen).

### Image processing

Following confocal imaging, the acquired images were subjected to processing and file-type conversion using ImageJ software (version 1.53t, National Institutes of Health, Bethesda, MD).

### Statistical analysis

**Fig. 1:**
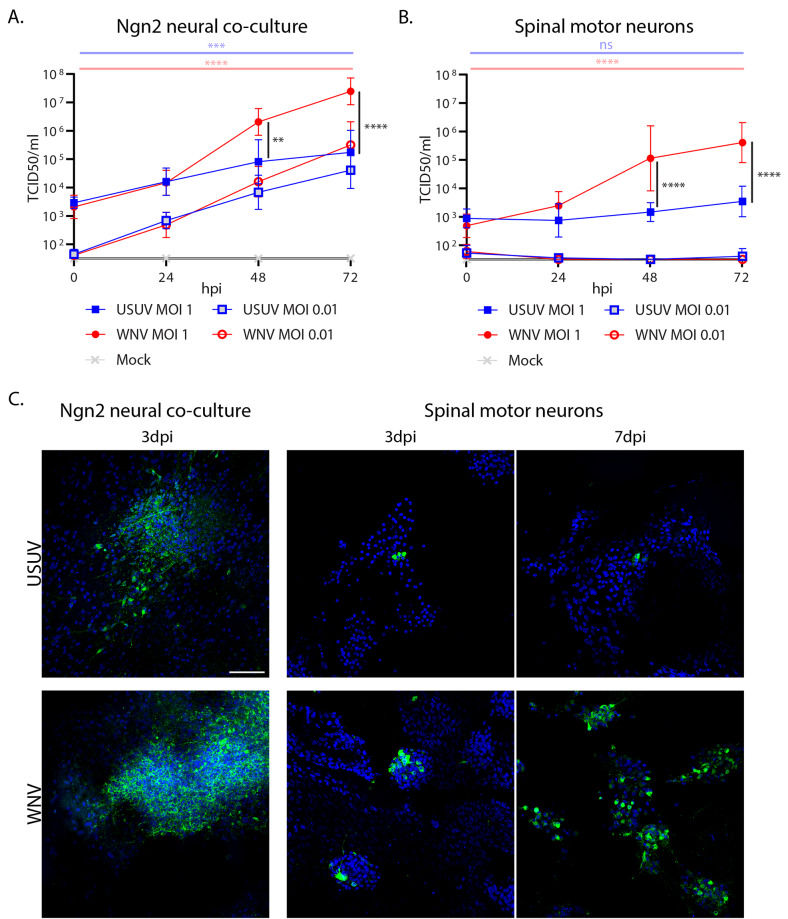
USUV can productively infect and spread within Ngn2 neural co-cultures but not motor neurons. **A**. Growth kinetics of USUV and WNV on iPSC-derived Ngn2 neural co-cultures infected at MOI 1 and 0.01. 3 replicates per condition, per experiment. *n* = 3. ** *p* = 0.0018. *** *p* = 0.0004. **** *p* < 0.0001. **B**. Growth kinetics of USUV and WNV on iPSC-derived motor neuron cultures infected at MOI 1 (*n* = 3) and 0.01 (*n* = 2). 3 replicates per condition, per experiment. Mean with SD. **** *p* < 0.0001. ns = non-significant. Horizontal blue line = USUV MOI 1 0hpi vs. USUV MOI 1 72hpi. Horizontal red line = WNV MOI 1 0hpi vs. WNV MOI 1 72hpi. Vertical black lines = USUV MOI 1 vs. WNV MOI 1. Data displayed has been log-transformed (Y = log[Y]). 2-way ANOVA with multiple comparison carried out on log-transformed data **C**. IF staining of viral envelope protein showing the distribution of USUV- and WNV-infected cells within iPSC-derived Ngn2 neural co-cultures and motor neuron cultures at 3 and 7 dpi. DAPI is shown in blue. Flaviviral envelope protein is shown in green. Scale bar represents 100 μm. Representative images of 3 experiments. An MOI of 1 was used for infection

Quantitative data were analysed and the statistical tests detailed in the figure legends were carried out using Prism 9.4.1 (GraphPad). As viral titres are presented on a logarithmic scale and have exponential growth, the raw TCID50/ml values obtained from titration were log-transformed (Y = log[Y]) using GraphPad Prism prior to statistical analysis (as presented in Fig. 1A-B and Fig. 4C-D) to aid in visualisation of the error bars and allow for better representation of the central point of data, as encouraged by Richardson et al. [32]. 2-way ANOVA with multiple comparison was employed to allow for comparison of all means of each different condition across time.

## Results

### USUV productively infects iPSC-derived Ngn2 neural co-cultures but not motor neurons

To identify and compare the tropism of USUV and WNV in cortical and motor neurons, we infected human iPSC-derived cortical neuron-astrocyte co-culture (Ngn2 neural co-culture) and spinal cord motor neuron models at a high (1) and low (0.01) multiplicity of infection (MOI). We found that both USUV and WNV could infect and replicate in the Ngn2 neural co-cultures at both MOIs (Fig. 1A), in absence of cytopathic effect. At MOI 0.01, USUV and WNV did not show significant differences in replication or peak titres, whilst at MOI 1, WNV grew to significantly higher titres than USUV after 24 hpi. Interestingly, in motor neurons, only WNV was able to productively replicate at an MOI of 1 (Fig. 1B), showing significantly increased titres at 48 hpi and 72 hpi compared with USUV. At an MOI of 0.01, WNV did not show increasing titres. USUV did not show significantly increased titres above input at any time point for either high or low MOI, compared to time point 0 hpi.

To investigate the distribution of infection we performed immunofluorescent (IF) staining to detect flaviviral envelope protein. At 3 days post-infection (dpi), large foci of infected cells could be identified in the Ngn2 neural co-cultures in both the USUV- and WNV-infected model systems. Both USUV and WNV primarily infected neurons, as identified by positive staining for neuronal marker MAP2 (Fig. S3A-B). In the motor neurons, USUV-infected cultures showed sparse positivity for viral envelope at 3 dpi and 7 dpi. In WNV-infected motor neuron cultures, we observed scattered foci of infection at 3 dpi, which increased in number at 7 dpi (Fig. 1C). Overall, these data show that WNV replicates efficiently in both Ngn2 and motor neurons, whereas USUV only productively replicates in Ngn2 neural co-cultures. This difference did not stem from a differing ability to attach to/enter motor neurons, as we saw no significant difference in the amount of viral RNA between USUV and WNV-exposed cells immediately after the inoculation period (Fig. S4).

### USUV, but not WNV, induces robust interferon responses in motor neurons

**Fig. 2:**
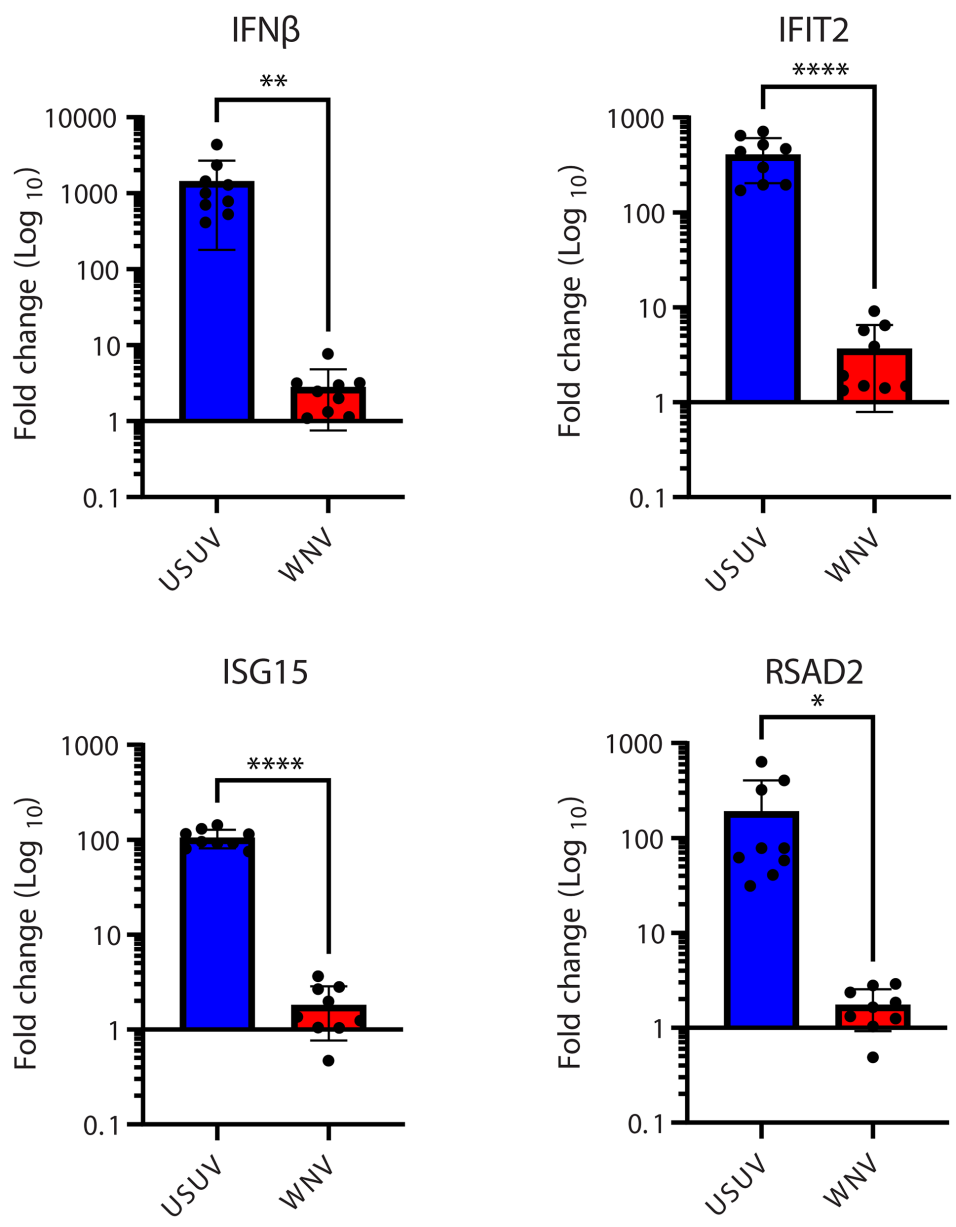
USUV induces a robust interferon response in motor neurons at 24hpi, but WNV does not. Fold change over mock of: (**A**) IFNβ. (**B**) IFIT2. (**C**) ISG15 and (**D**) RSAD2 in iPSC-derived motor neuron cultures infected with USUV or WNV at an MOI of 10. All cultures were lysed at 24hpi and ISG transcripts were quantified by qPCR. 4 culture wells were pooled per replicate for a total of 3 replicates for analysis, per condition, per experiment. *n* = 3. Mean with SD. * *p* = 0.0182. ** *p* = 0.0035. **** *p* < 0.0001. Unpaired t-test

As we observed no difference in attachment/entry, we then hypothesised that the inability of USUV to replicate in motor neurons may stem from a different induction of the intrinsic immune response at early time points, compared to WNV. To characterise the initial response of the motor neurons to infection with USUV or WNV, we quantified the expression levels of interferon beta (IFN-β) and a panel of 3 interferon (IFN) stimulated genes (ISGs), which play an important role in restricting virus replication. Neither USUV nor WNV led to a substantial increase in IFN-β or ISG transcripts compared with mock at 12 hpi (Fig. S5A-D). However, at 24 hpi, USUV infection led to significant increases in IFN-β and ISG levels (expressed as fold change over mock) compared with WNV, with an average increase of 1433-fold for IFN-β (Fig. 2A), 404-fold for IFIT2 (Fig. 2B), 104-fold for ISG15 (Fig. 2C) and 190-fold for RSAD2 (Fig. 2D). Comparatively, for WNV, no significant IFN responses were detected. These data show a clear induction of the IFN response following USUV infection but not WNV infection, indicating that the IFN response restricts USUV replication in motor neurons.

### Blockade of the intrinsic immune response allows for USUV infection of motor neurons

**Fig. 3:**
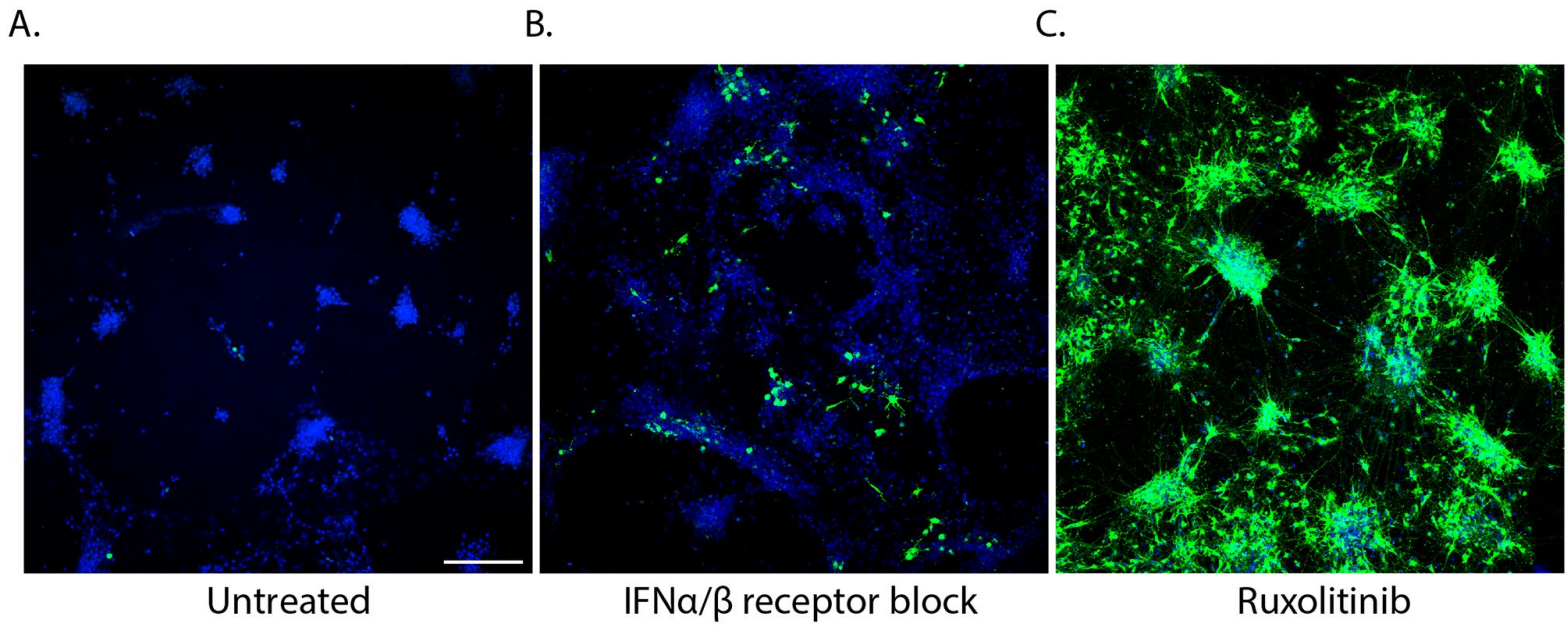
Inhibition of the antiviral response enables spread of USUV infection in motor neurons. IF staining of USUV envelope protein to show the distribution of infection at 72 hpi in (**A**) untreated cultures, (**B**) cultures pre-treated with 5 μg/ml of human type 1 IFN receptor neutralising antibody for 2 h, and (**C**) cultures pretreated with 8μM of the JAK1/2 inhibitor, Ruxolitinib for 2 h. Representative images of 3 experiments. An MOI of 1 was used for infection. DAPI is shown in blue. Flaviviral envelope protein is shown in green. Scale bar represents 200 μm

The observed differences in induction of the intrinsic antiviral response led us to hypothesise that blockade of this response would allow for infection of the motor neurons by USUV. To investigate this, we employed two methods of inhibition of the antiviral response. First, we aimed to determine the significance of type 1 IFNs in the observed difference in susceptibility by treating cultures with an antibody that neutralizes type 1 IFN receptors. Compared with the untreated condition (Fig. 3A), we found that blocking type 1 IFN receptors led to an increased number of infected cells (Fig. 3B). We then aimed to more comprehensively inhibit the intrinsic immune response via treatment with Ruxolitinib, a JAK1/2 inhibitor which inhibits the production of ISGs via blockade of the IFN-signalling cascade. This inhibition resulted in widespread infection of up to 100% of cells (Fig. 3C). Overall, these results show that inhibition of the type I IFN response enables infection of motor neurons with USUV.

### Both USUV and WNV can undergo axonal transport

**Fig. 4:**
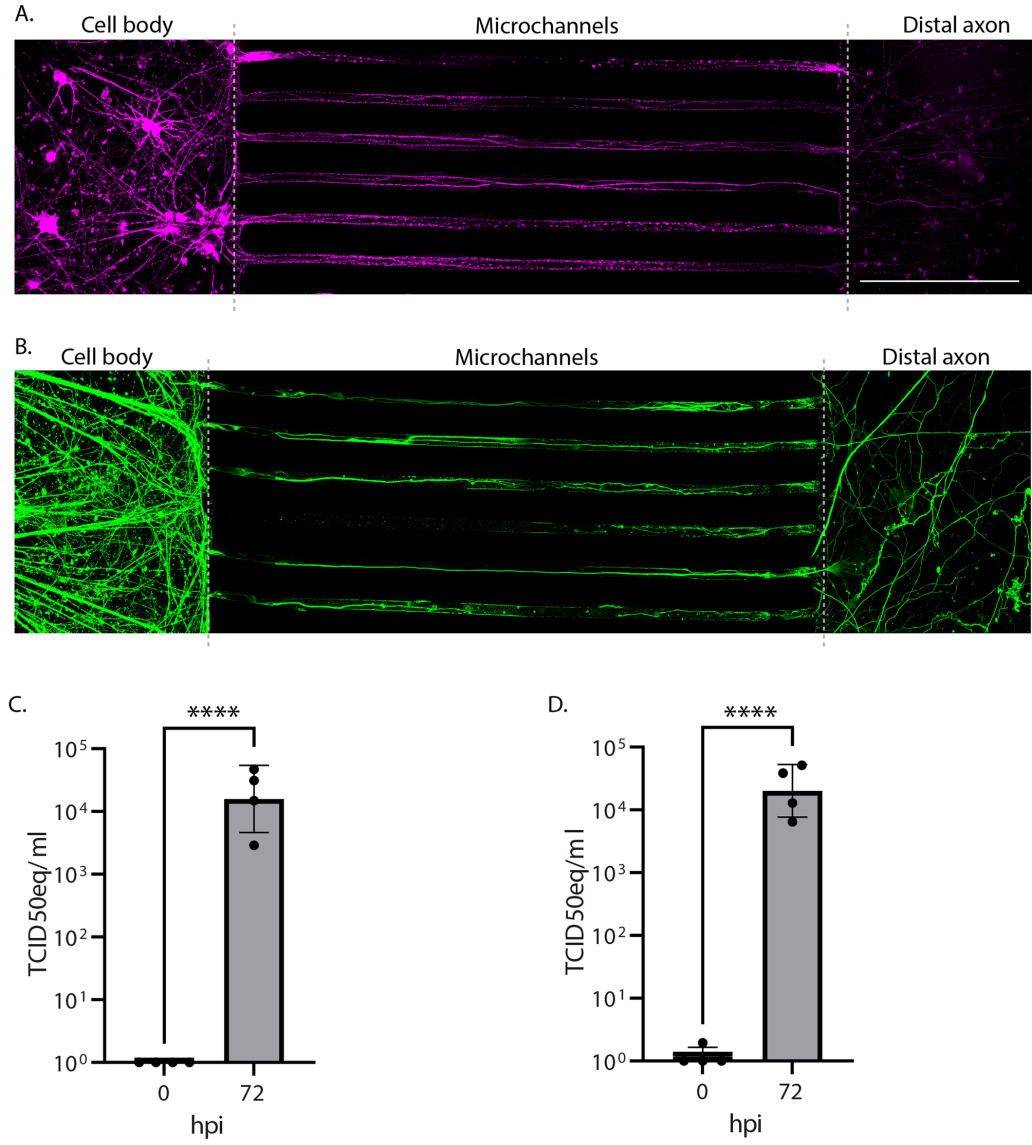
WNV and USUV can be transported along axons within a fluidically isolated, compartmented motor neuron culture system. IF staining of (**A**) dendrite marker, MAP2, and (**B**) axon marker, NF200, to show penetration of neurites of the iPSC-derived motor neurons from the cell compartment, through the microchannels and into the distal axon compartment of the compartmented culture system. Dotted lines indicate the separation of the compartments. Scale bar represents 200 μm. (**C**) WNV titre equivalents in the distal axon compartment of the compartmented culture system at 0 and 72 hpi following inoculation at MOI 10. (**D**) USUV titre equivalents in the distal axon compartment of the compartmented culture system at 0 and 72 hpi following inoculation at MOI 10. USUV-infected cultures were pretreated with 8μM of the JAK1/2 inhibitor, Ruxolitinib, 2 h prior to infection. RT-qPCR was used to quantify presence of viral genome, which was compared against a standard curve of diluted virus stock to obtain TCID50eq values. 2 replicates per condition, per experiment. *n* = 2. Mean with SD. **** *p* < 0.0001. Data displayed has been log-transformed (Y = log[Y]). Unpaired t-test carried out on transformed data

To study the transportation of virus along motor neurons, we developed a fluidically isolated compartmented culture system. Staining for the dendrite microtubule-associated protein, MAP2 (Fig. 4A), and the axonal neurofilament, NF200 (Fig. 4B), showed that at 2 weeks post-seeding, the motor neurons had extended dendrites and axons through the microchannels into the adjacent compartment. To validate the occurrence of axonal transport in this model, we tested whether virus could be transported along axons in the microchannels from the fluidically isolated cell body compartment into the distal axon compartment. Following inoculation, no viral genome could be detected by qPCR in the distal axon compartment, while at 72 hpi a significant increase in the presence of WNV genome was detected in the distal axon compartment compared with 0 hpi (Fig. 4C). We then studied whether USUV could also be transported via axonal transport when given the opportunity to productively infect via inhibition of the intrinsic immune response. After pre-treatment with Ruxolitinib, USUV showed comparable results to WNV, with a significant increase in the presence of viral genome in the distal axon compartment at 72 hpi, compared with 0 hpi (Fig. 4D). Overall, we observed no difference in the ability of USUV and WNV to be transported within a compartmented culture system of human iPSC-derived motor neurons.

## Discussion

WNV and USUV are both considered neurotropic viruses. However, there is an apparent difference in the capacity of these viruses to induce severe neurological disease in humans. In this study, we aimed to uncover key mechanisms underlying this difference by identifying the conditions under which USUV can infect and replicate within Ngn2 neural co-cultures and spinal cord motor neurons, to model infection of the brain and the cellular components of a route of transneural neuroinvasion. We showed efficient replication and dissemination of USUV within human iPSC-derived Ngn2 neural co-cultures, which supports previous findings in which USUV has been shown to replicate in murine neurons, human neuronal stem cells and human astrocytes [33]. These results suggest that once within the CNS, USUV can replicate and spread efficiently, albeit to a slightly lesser degree compared with WNV. Therefore, we hypothesised that the low incidence of USUV-associated neurological disease and motor deficit might not stem from a reduced ability of USUV to infect the cells of the brain but instead result from a reduced ability of USUV to gain access to the CNS. WNV has been indicated to use a transneural route of invasion in both an in vivo hamster model [14, 15] and within in vitro rat motor neurons [14], and cases of fatal human WNV infections have reported severe motor deficits and shown the presence of viral antigen in the spinal cord and basal regions of the brain [10, 34–36] which may be suggestive of an ascending route of invasion. Previous work has also described a preference for transport of WNV along motor neurons, rather than sensory neurons, to the cell bodies present in the ventral horn of the spinal cord [15]. Similarly, in a suckling mouse model of USUV infection, neuronal death and the presence of viral antigen within the ventral horn of the spinal cord was identified [37], suggesting that USUV may exhibit a similar tropism for spinal motor neurons as WNV.

However, in the current study, we showed that whilst WNV could productively infect and spread within in vitro cultures of human iPSC-derived spinal cord motor neurons, USUV could not. This difference did not appear to stem from an inability of USUV to bind or infect the motor neurons, but from an inability to replicate and disseminate. USUV has previously been found to have an increased sensitivity to type I and type III IFN responses, compared with WNV [38], and the resistance of WNV to type I interferon signalling was identified as a key determinant in its replication fitness and virulence [39]. Therefore, we investigated potential differences in the induction of the intrinsic response between USUV and WNV. The intrinsic immune response acts to promote an antiviral state within a host cell that directly restricts viral replication, assembly and spread. This occurs via recognition of viral components leading to production and secretion of IFNs which, via JAK/STAT signalling, results in transcription of hundreds of ISGs both within the infected cells and the surrounding uninfected cells [40]. WNV employs a variety of immune evasive strategies, some of which are dependent upon non-structural proteins such as NS5, which has been shown to act as a potent JAK/STAT inhibitor [41]. We observed a substantial induction of a subset of key ISGs [42–44] by USUV at early-time points and a stark increase in infection percentage upon treatment of motor neurons with the JAK/STAT inhibitor, Ruxolitinib. These results could suggest a reduced efficacy of JAK/STAT inhibition by USUV non-structural proteins compared with WNV, leading to a more effective control of viral replication and dissemination by the IFN response. Overall, these findings tie into the in vivo study discussed previously in which USUV-infected spinal cord motor neurons were identified, as suckling mice have a dampened IFN response compared with adult mice [45]. Further, WNV has been demonstrated to evade initial detection by host cells via interaction of WNV NS1 with host pattern recognition receptors, such as RIG-I [46, 47] and TLR3 [48], which may explain the lack of ISG induction we observed upon WNV infection of motor neurons. Future work should focus on using this model to study the mechanisms of induction, or inhibition, of the intrinsic immune response by USUV and WNV. Such work could include the development of chimeric viruses via exchange of non-structural proteins of USUV and WNV, allowing for identification of the relative role of each protein in the modulation of the intrinsic response.

Interestingly, our observation that a type I IFN receptor blocking antibody allowed for more widespread USUV infection of motor neurons may be highly relevant for the clinical picture of USUV-related disease when considering the recent findings that 40% of a cohort of 441 patients with WNV neuroinvasive disease [49] and 3 out of 8 patients with severe adverse reaction to the live-attenuated vaccine against yellow fever virus, another flavivirus, had auto-antibodies to type I IFNs [50], indicating the profound importance of IFN in the control of flaviviruses, and the risk resulting from disruption of this response. Further, using a compartmented culture system, we showed that in the absence of an interferon response, there is no intrinsic difference in the ability of USUV and WNV to be transported along motor neurons. Therefore, our data suggests that patients with an impaired IFN response may be at an increased risk of neurological disease and motor deficit resulting from infection by USUV. To confirm this extrapolation and expand our understanding of the risk factors underlying the development of USUV-associated neurological disease, the presence of anti-IFN auto-antibodies in neurological USUV disease cases should be determined. As USUV infection is not a mandatory notifiable disease in the European Union [5], a continued concerted effort between clinicians and researchers is required to ensure in-depth investigation and reporting of USUV-associated disease. This information would allow for validation of our findings using clinicopathological observations from cases of human infection. In the meantime, confirmation of our data in multiple iPSC donor lines would further strengthen the data obtained in this study. Furthermore, whilst we have established a compartmented culture system, additional optimisations are required to model retrograde transport and identify the exact transport machinery involved in the transport of USUV and WNV towards and within the CNS.

## Conclusions

Our data suggest that the reduced occurrence and severity of neurological disease resulting from infection with USUV compared with WNV is due to a difference in induction of the IFN response. In healthy individuals, USUV may be unable to infect motor neurons and must instead rely on alternate routes to access, damage and disseminate within the CNS. However, our data suggests that patients with underlying deficiencies in the IFN response are at risk of developing neurological disease following USUV infection.

## Electronic supplementary material

Below is the link to the electronic supplementary material.

Supplementary Material 1

Supplementary Material 2

## Data Availability

All data referred to in this manuscript is included in the manuscript and supplementary materials. The datasets used and analysed during the current study are available from the corresponding author upon reasonable request. An MTA is in place with the Gladstone Institute (Bruce Conklin) for the WTC-11 cell line (UBMTA 2023-0123).

## Abbreviations

CNS: Central nervous system
CPE: Cytopathic effect
dpi: Days post infection
IF: Immunofluorescent
IFN: Interferon
iPSC: Induced pluripotent stem cell
ISG: Interferon stimulated gene
MOI: Multiplicity of infection
Ngn2: Neuronal determinant neurogenin-2
TCID50: 50% tissue culture infective dose
USUV: Usutu virus
WNV: West Nile virus
