# Supplementary material for: Differential susceptibility of human motor neurons to infection with Usutu and West Nile virus
Source: J Neuroinflammation. 2024 Sep 27;21:236. doi: 10.1186/s12974-024-03228-y (PMC11437828; doi:10.1186/s12974-024-03228-y)
Supplement: Supplementary file 1 — Supplementary Material 1 [file 12974_2024_3228_MOESM1_ESM.pdf]

Supplementary methods:

Quantitative PCR characterization of motor neuron cultures

RNA was isolated from iPSCs and day 21 iPSC-derived spinal motor neuron cultures using the High Pure RNA isolation kit (Roche). The concentration of RNA was determined using a NanoDrop spectrophotometer. 500ng of RNA was reverse transcribed into cDNA using the SuperScript III reverse transcriptase (Invitrogen) according to the manufacturer's protocol. Gene expression was determined with gene specific primers **(Table S1.)** using SYBR® Green PCR Master Mix (Life technologies). Gene of interests were normalized to TATA-binding protein.

Supplementary table 1: Sequences of primers used for characterisation of iPSC-derived spinal cord motor neuron cultures

Gene	Forward primer	Reverse primer
**OCT4**	GATGGCGTACTGTGGGCCC	TGGGACTCCTCCGGGTTTTG
**SOX2**	GGGAAATGGGAGGGGTGCAAAAGAGG	TTGCGTGAGTGTGGATGGGATTGGTG
**NANOG**	CAGCCCTGATTCTTCCACCAGTCCC	TGGAAGGTTCCCAGTCGGGTTCACC
**HB9**	GCACCAGTTCAAGCTCAACA	TTTGCTGCGTTTCCATTTC
**ISL1**	TGTTTGAAATGTGCGGAGTG	GCATTTGATCCCGTACAACC
**CHAT**	TGAGTACTGGCTGAATGACATG	AGTACACCAGAGATGAGGCT
**PAX6**	GCCCTCACAAACACCTACAG	TCATAACTCCGCCCATTCAC
**TATA-BP**	GGGGAGCTGTGATGTGAAGT	CCAGGAAATAATTCTGGCTCA
