# Supplementary material for: Differential susceptibility of human motor neurons to infection with Usutu and West Nile virus
Source: J Neuroinflammation. 2024 Sep 27;21:236. doi: 10.1186/s12974-024-03228-y (PMC11437828; doi:10.1186/s12974-024-03228-y)
Supplement: Supplementary file 2 — Supplementary Material 2 [file 12974_2024_3228_MOESM2_ESM.pdf]

Figure S1

A.

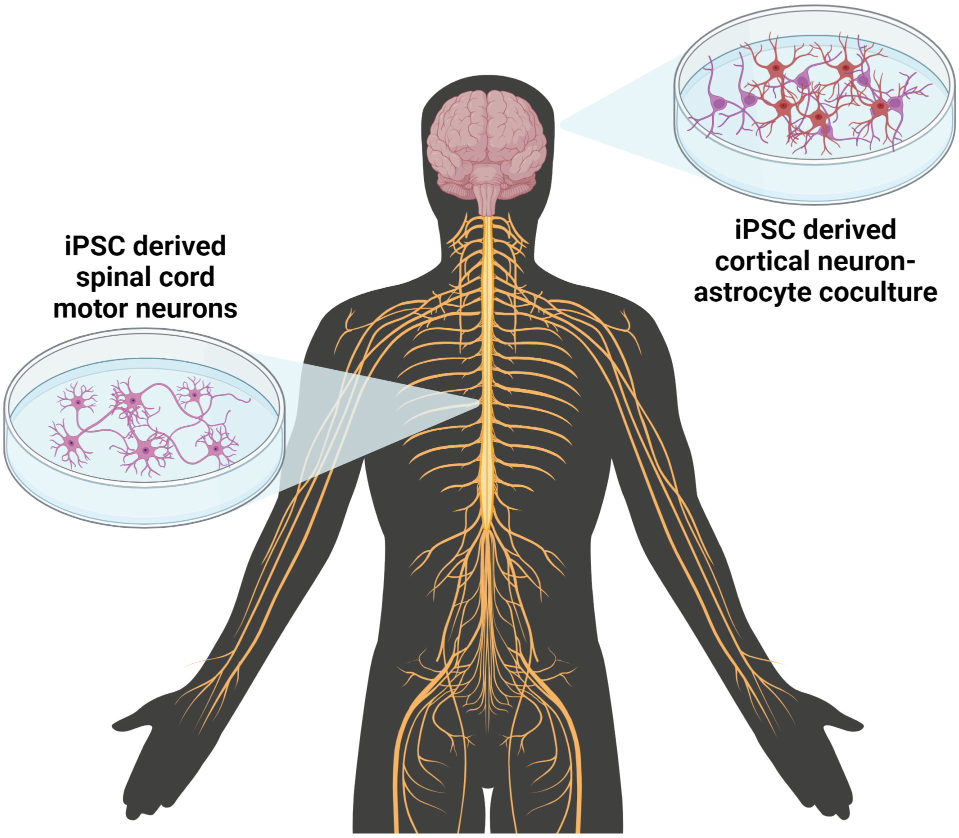

B.

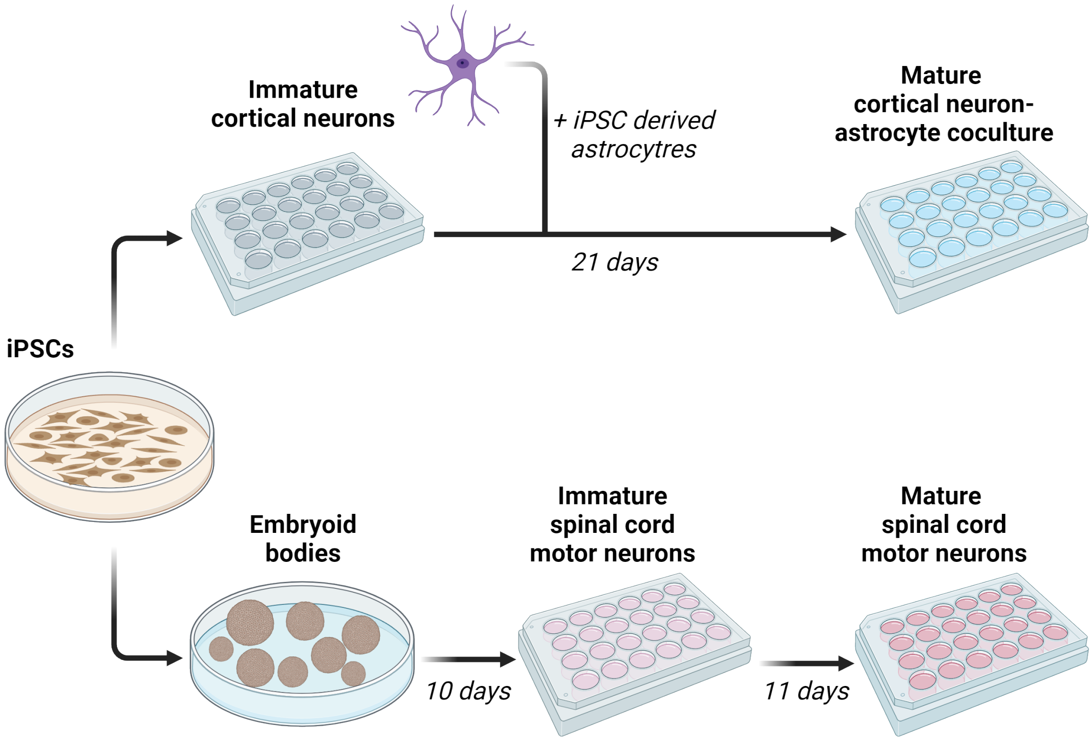

Supplementary figure 1. Models and methods of iPSC culture. A. Regions the neuronal subtypes used in this study are intended to model. **B.** Simplified overview of iPSC-derived Ngn2 neural co-culture and spinal cord motor neuron differentiation. Created with biorender.

A.

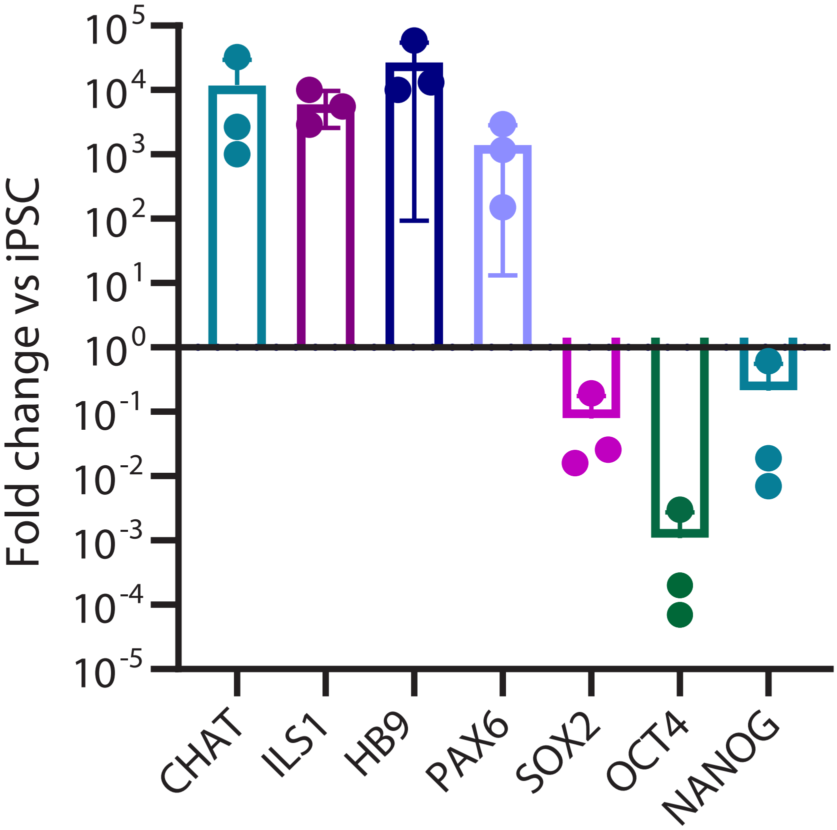

B.

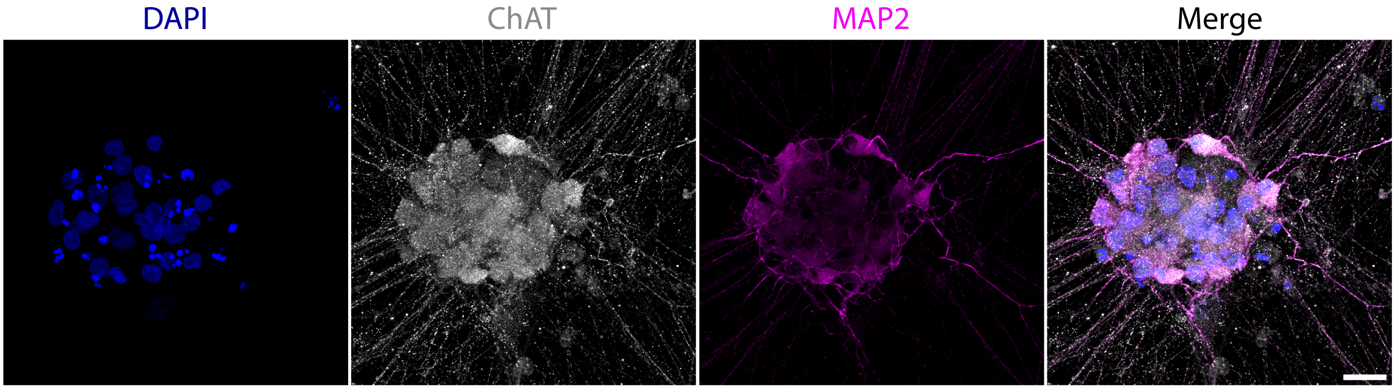

Supplementary figure 2. iPSC-derived spinal cord motor neurons express the expected motor neuron specific markers. A. qPCR of motor neuron specific (ChAT, ILS1, HB9) and progenitor (SOX2, OCT4, NANOG) marker expression, displayed as fold change of day 21 iPSC derived motor neurons compared to iPSC cultures. n=3. Data derived from 3 separate differentiations. **B.** Representative IF staining of ChAT in iPSC derived motor neuron cultures. Scale bar represents 20µm.

Figure S3

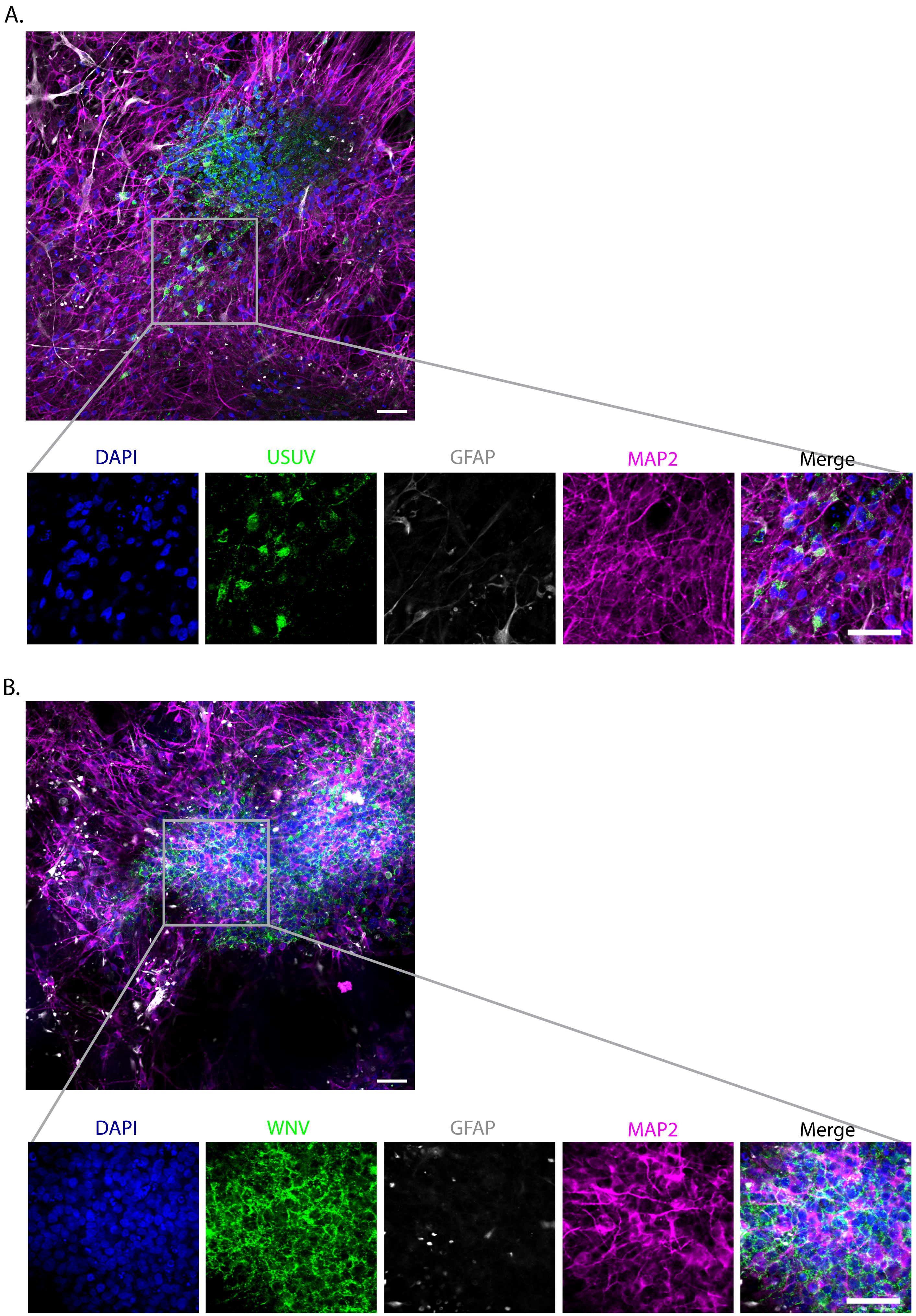

Supplementary figure 3. USUV and WNV primarily infect neurons of the iPSC-derived Ngn2 neural co-culture system. A. IF staining of USUV and **B.** WNV envelope protein within MAP2 positive neurons present in the iPSC-derived Ngn2-neural co-culture system at 72 hpi following MOI 1 infection. Cultures were infected after a minimum of 21 days of differentiation. DAPI is shown in blue. Flaviviral envelope protein is shown in green. GFAP, an astrocyte marker, is shown in white. MAP2, a neuronal marker, is shown in pink. Representative image from 3 experiments. Scale bars represent 50μm.

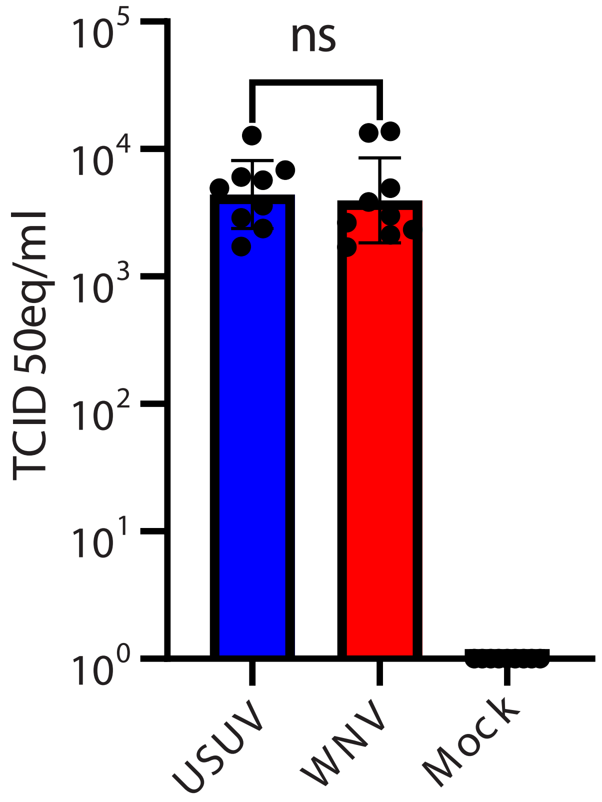

Supplementary figure 4. USUV and WNV do not show a difference in entry/attachment. USUV and WNV titre equivalents following inoculation, washing, and lysis of iPSC-derived motor neuron cultures infected at MOI 1. qPCR was used to quantify the presence of viral genome in cell lysates, which was compared against a standard curve of diluted virus stock to obtain TCID50eq/ml values. 3 replicates per condition, per experiment. n=3. Mean with SD. ns = non-significant. Unpaired t-test.

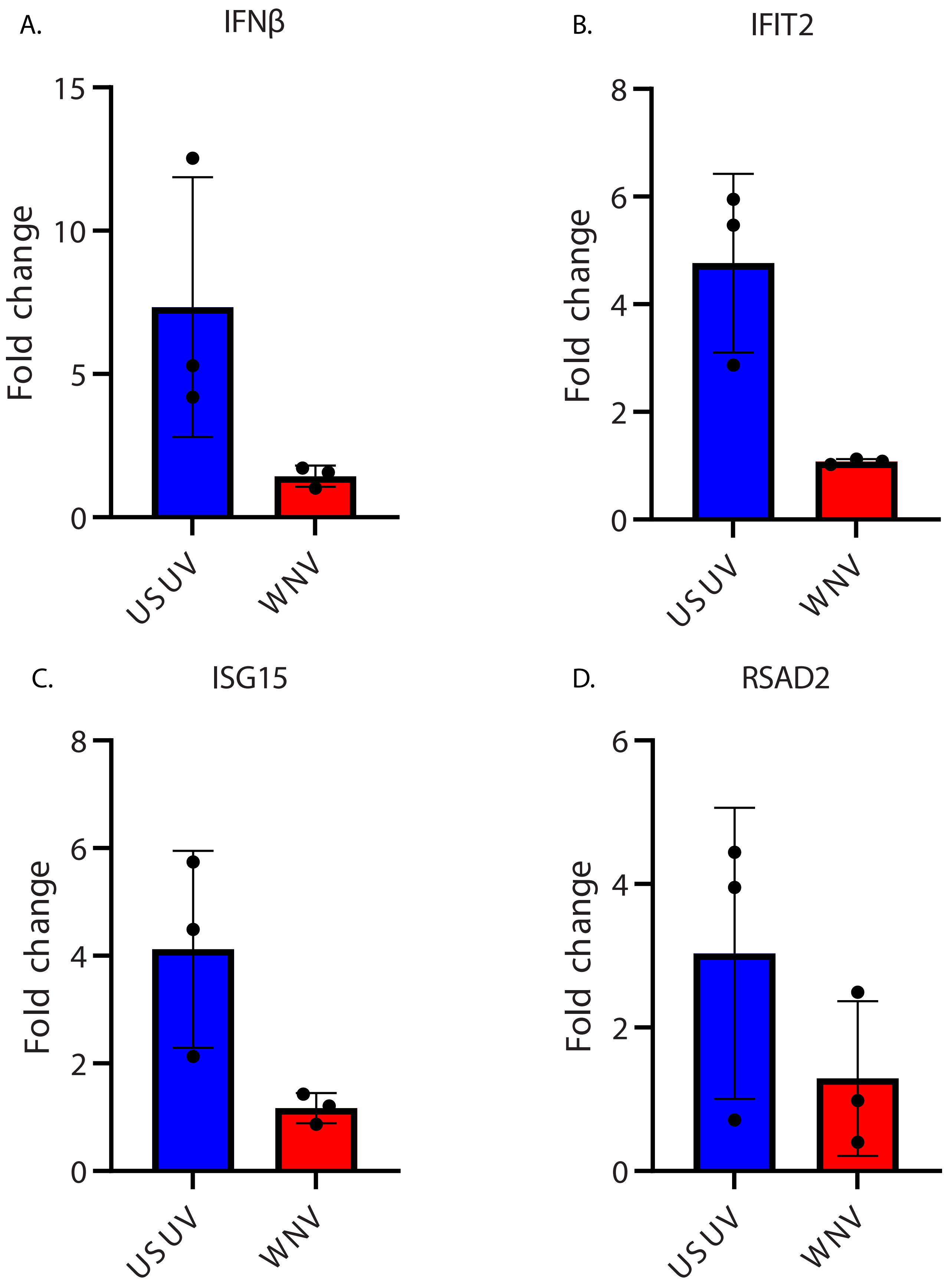

Supplementary figure 5. The interferon response induced in infected motor neuron cultures is limited at 12hpi. Fold change over mock of: **A.** IFNβ. **B.** IFIT2. **C.** ISG15. **D.** RSAD2 in iPSC-derived motor neuron cultures infected with USUV or WNV at an MOI of 10. Cultures were lysed at 12hpi and ISG transcripts were quantified by qPCR. 4 culture wells were pooled per replicate for a total of 3 replicates per condition for analysis. n=1. Mean with SD.
